# Single‐Cell RNA‐Seq of T Cells in B‐ALL Patients Reveals an Exhausted Subset with Remarkable Heterogeneity

**DOI:** 10.1002/advs.202101447

**Published:** 2021-08-08

**Authors:** Xiaofang Wang, Yanjuan Chen, Zongcheng Li, Bingyan Huang, Ling Xu, Jing Lai, Yuhong Lu, Xianfeng Zha, Bing Liu, Yu Lan, Yangqiu Li

**Affiliations:** ^1^ Department of Hematology First Affiliated Hospital Jinan University No. 601 West of Huangpu Avenue Guangzhou 510632 China; ^2^ Key Laboratory for Regenerative Medicine of Ministry of Education Institute of Hematology School of Medicine Jinan University Guangzhou 510632 China; ^3^ State Key Laboratory of Experimental Hematology Institute of Hematology Fifth Medical Center of Chinese PLA General Hospital Beijing 100071 China; ^4^ Department of Clinical Laboratory First Affiliated Hospital School of Medicine Jinan University No. 601 West of Huangpu Avenue Guangzhou 510632 China

**Keywords:** B cell‐acute lymphoblastic leukemia, heterogeneity, single‐cell RNA sequencing, T cells

## Abstract

Characterization of functional T cell clusters is key to developing strategies for immunotherapy and predicting clinical responses in leukemia. Here, single‐cell RNA sequencing is performed with T cells sorted from the peripheral blood of healthy individuals and patients with B cell‐acute lymphoblastic leukemia (B‐ALL). Unbiased bioinformatics analysis enabled the authors to identify 13 T cell clusters in the patients based on their molecular properties. All 11 major T cell subsets in healthy individuals are found in the patients with B‐ALL, with the counterparts in the patients universally showing more activated characteristics. Two exhausted T cell populations, characterized by up‐regulation of *TIGIT*, *PDCD1*, *HLADRA*, *LAG3*, and *CTLA4* are specifically discovered in B‐ALL patients. Of note, these exhausted T cells possess remarkable heterogeneity, and ten sub‐clusters are further identified, which are characterized by different cell cycle phases, naïve states, and *GNLY* (coding granulysin) expression. Coupled with single‐cell T cell receptor repertoire profiling, diverse originations of the exhausted T cells in B‐ALL are suggested, and clonally expanded exhausted T cells are likely to originate from CD8^+^ effector memory/terminal effector cells. Together, these data provide for the first‐time valuable insights for understanding exhausted T cell populations in leukemia.

## Graphical abstract

Single‐cell RNA sequencing analyses reveal common activation across T cell subsets under B cell‐acute lymphoblastic leukemia. Exhausted T cells specifically exist in patients, showing remarkable heterogeneity in molecular characteristics and extensive diversity in clonal derivation. In this study, valuable insights are provided for deeply understanding exhausted T cell populations in leukemia.

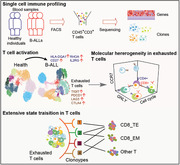

## 1 Introduction

B cell‐acute lymphoblastic leukemia (B‐ALL) is a clonal hematopoietic disease characterized by the abnormal proliferation and accumulation of B‐lymphoid progenitor cells.^[^ ^1^, ^2^ ^]^ Although most patients initially respond to chemotherapy, ≈60% relapse, and only 30% survive within 5 years; thus, overall anti‐tumor responses remain limited.^[^ ^3^ ^]^ Current chemotherapies aim at killing abnormal leukemia cells while inevitably damaging normal cells and carrying significant risk of long‐term toxicities. Therefore, new strategies using fewer toxic elements are urgently needed. Over the past decade, immune‐based approaches such as monoclonal antibody therapy and checkpoint inhibitor therapy have emerged.^[^ ^4^ ^]^ T cells with chimeric antigen receptors (CARs) have led to impressive responses and encouraging outcomes in patients with hematological malignancies.^[^ ^5^, ^6^ ^]^ Furthermore, it is increasingly appreciated that the immune microenvironment governs the strength of the anticancer response following immune therapies.^[^ ^7^, ^8^ ^]^ For instance, the efficacies of CAR‐T cell therapy are not uniform among patients and closely related to the activity of derived T cells. Therefore, deepening the understanding of inhibition states and the functional heterogeneity of T cells in patients will be paramount to revealing new therapeutic targets.

Currently, prognostic factors for leukemias depend on their risk stratification, including the genomic features of leukemia cells and clinical characteristics, for example, age, the count of white blood cell (WBC) at diagnosis, and response to chemotherapy.^[^ ^9^ ^]^ Whether the properties of the host immune system affect disease risk or outcome in leukemia is unknown. Because the immune microenvironment is highly complex, evaluation of clinical outcome requires deep understanding of the T cells underlying the leukemia. The efficacy of many checkpoint inhibitors largely depends on their ability to reduce CD8^+^ cytotoxic T cells in the microenvironment, whose cytotoxic ability may be rendered inactive by reaching a T cell dysfunction state called exhaustion.^[^ ^10^ ^]^ Exhausted T cells could be defined by sustained expression of inhibitory receptors (*CTLA‐4*, *PD‐1*, *TIGIT*, *LAG3*, *TIM3*). However, exhausted T cells were previously associated with a loss in proliferative capacity, and recent studies have provided evidence for a highly proliferating population within human tumors.^[^ ^2^, ^11^ ^]^ Therefore, a thorough investigation of the accumulated T cell exhaustion in leukemia will provide a better strategy for leukemia treatment and outcome prediction.

With the progress of single‐cell RNA sequencing (scRNA‐seq), a number of recent single‐cell studies have illustrated the immune microenvironment of human tumors.^[^ ^12^, ^13^, ^14^ ^]^ In this study, the scRNA‐seq analysis of 28 842 single T cells isolated from healthy individuals and B‐ALL patients enabled us to simultaneously study their transcriptomes and T cell receptor (TCR) sequences. We identified 13 unique T cell subsets with distinct molecular properties. Signature genes for exhausted T cells were examined in detail, and these cells were mostly derived from CD8+ effector memory/terminal effector cells and clonally expanded in leukemia. These data provide valuable insights for understanding the exhausted populations in leukemia and can be used as a valuable resource for studying the basic characteristics of T cells in leukemia and potentially guiding effective immunotherapy strategies.

## 2 Results

### 2.1 Two Exhausted T Cell Clusters Were Specifically Identified in B‐ALL Patients

**Figure 1:**
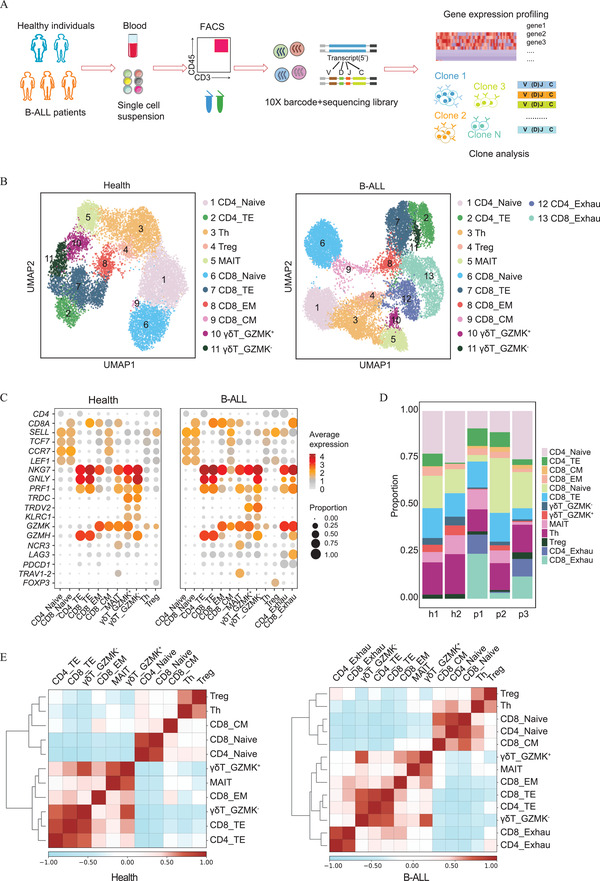
Transcriptomic clustering of CD45^+^CD3^+^ T cells from healthy individuals and B‐ALL patients. A) Schematic of the experimental design for scRNA‐seq. Peripheral blood mononuclear cells were collected from 2 healthy individuals and 3 B‐ALL patients. T cells with the immunophenotype CD45^+^CD3^+^ were sorted using Aria III and subjected to scRNA‐seq combined with single‐cell TCR sequencing using a 10×‐Based Genomics platform. B) UMAP projection of eleven T cell clusters of healthy individuals (left, 12 699 cells in total) and thirteen cell clusters of B‐ALL patients (right, 16 143 cells in total). Each dot corresponds to one single cell colored according to cell cluster. C) Dot plots show the average expression levels and cell expression proportions of selected nineteen T cell marker genes in the indicated clusters for healthy individuals (left) and patients (right). The colors represent the average expression levels, and dot sizes represent the percentage expression of selected genes in the indicated clusters. D) Stacked bar chart showing the constitution of thirteen T cell clusters in each healthy individual (abbreviated as “h1” and “h2”) and B‐ALL patient (abbreviated as “p1,” “p2,” and “p3”). E) Heatmaps showing the pairwise Pearson correlation coefficients of eleven or thirteen T cell clusters for HIs (left) and B‐ALL patients (right). The clusters were ordered by hierarchical clustering the corresponding correlation matrix.

Although T cells play critical roles in the acute leukemia environment, their heterogeneity and impact on leukemia progression remain insufficiently characterized.^[^ ^15^ ^]^ To better understand the T cell immune state of B‐ALL, we constructed transcriptomic maps for both the healthy and B‐ALL disease states. Peripheral blood samples from two healthy individuals and three treatment‐naïve B‐ALL patients were collected, and then the CD45^+^CD3^+^ cell populations purified by fluorescence‐activated cell sorting (FACS) were subjected to scRNA‐seq and TCR sequencing using the 10× Genomics platform (**Figure** **1**A). After strict quality control, transcriptomic maps of 12 669 and 16 143 T cells, of which 3586 and 14 550 cells had paired TCR clonotypes, were retained for the healthy and B‐ALL states, respectively (Figure S1A and Table S1, Supporting Information). On average, 1590 genes and 5439 unique molecular identifiers (UMIs) were detected in a single T cell (Figure S1B,C, Supporting Information).

Unsupervised clustering analysis grouped single T cells into 11 clusters in healthy individuals and 13 clusters in B‐ALL patients. The distinct expression patterns of canonical T cell markers allowed us to annotate these clusters with well‐known T cell subtypes (Figure 1B,C), which were further confirmed using the cell type identification package‐“SingleR,” coupled with reference datasets (see Experimental Section for detail)^[^ ^16^ ^]^ (Figure S1D,E, Supporting Information). Naïve CD4^+^ T cells (CD4_Naive) and naïve CD8^+^ T cells (CD8_Naive) were two dominant clusters characterized by the high expression of *SELL, TGF7*, *CCR7*, and *LEF1* (also known as “naïve” marker genes).^[^ ^17^ ^]^ These two clusters carried low levels of cytokines and effector genes. Central memory T cells (CD8_CM), terminal effector T cells (CD4_TE and CD8_TE), and effector memory T cells (CD8_EM) were all characterized by low levels of naïve markers. However, the expression of cytotoxic markers (*NKG7*, *GNLY*, *PRF1*, *GZMK*, and *GZMH*) was low in CD8_CM and CD8_EM but high in CD4_TE and CD8_TE. These two clusters are commonly associated with T cells with effector function. Mucosal‐associated invariant T cells (MAITs) were characterized by predominantly bearing semi‐invariant TCR alpha chains with TRAV1‐2 and specific expression of *SLC4A10*, *RORC*, and *ZBTB16*.^[^ ^18^ ^]^ Both *γδ* T_GZMK^+^ and *γδ* T_GZMK^−^ cells were characterized by the expression of *TRDC*, *TRDV2*, and *KLRC2* with *GZMK* preferentially expressed in some *γδ* T cells as previously reported.^[^ ^19^ ^]^ Markedly high expression of cytotoxic activity related genes (*NKG7*, *GNLY*, and *GZMH*) was observed in MAIT, *γδ* T_GZMK^+^, and *γδ* T_GZMK^−^ cells. This observation is consistent with previous reports that MAIT and *γδ* T cells act as a first line of defense, and they possess the potential to kill leukemia cells.^[^ ^20^ ^]^ Treg cells were characterized by high expression of the Treg signature genes *Foxp3, IKZF2*, *IL2RA*, and *CTLA4* as well as co‐stimulatory markers, including *CD28* and *ICOS*. Th 1, Th 2, and Th 17 cells were all clustered as “T helper cells (Th cells)” due to their transcriptome similarities and indistinguishability. Specifically, two exhausted T cell clusters (CD4_Exhau and CD8_Exhau) were shown in B‐ALL patients, which were characterized with high levels of the exhaustion markers *LAG3*, *PDCD1, TIGIT*, and *HAVCR2* ^[^ ^21^ ^]^ (Figure 1C; Figures S1F,G, S2A,B, and Table S2, Supporting Information). Eleven T cell clusters were stably expressed in both healthy and B‐ALL samples, and two exhausted T cell clusters were patient‐specific (Figure 1D/Figure S2C, Supporting Information). This phenomenon agreed with previous findings in solid tumors.^[^ ^22^, ^23^ ^]^

Pearson correlation analysis was performed to delineate the relationship between T cell clusters in healthy individuals and B‐ALL samples (Figure 1E). It was found that the naïve, effector, and exhaustion T cell subtypes were clustered together, irrespective of CD4 or CD8 subtype. A close relationship between Treg and Th cells was shown, indicating a few similar genes were shared (*LTB*, *ITGB1*, and *PLP2*). CD4_Naive, CD8_Naive, and CD8_CM cells were close in position, as they all expressed the naïve marker genes. GZMK^−^ *γδ* T cells had a similar transcriptome as GZMK^+^ *γδ* T cells; however, they clustered with terminal effector subtypes (CD4_TE and CD8_TE). Moreover, MAIT cells were clustered with GZMK^+^ *γδ* T cells. All of these T cell clusters exert a direct cytotoxic effect inside the body. Last, two exhausted clusters (CD4_Exhau and CD8_Exhau) distinctly clustered together in B‐ALL patients. Based on these results, we can use the transcriptomically identified T cell clusters for in‐depth analysis of potential mechanisms that give rise to the new atlas of T cell clusters in B‐ALL blood.

B‐ALL is a B‐cell malignancy, which is associated with profound alterations and defects in the immune system, invading both peripheral blood and bone marrow. Considering the importance of bone marrow as another immunological environment, we next explored whether the proportion of exhausted T cells was comparable between peripheral blood and bone marrow, by re‐analyzing the scRNA‐seq datasets from GEO of a previous study that involved both peripheral blood and bone marrow of B‐ALL patients to recognize different T cell clusters (Figure S3A,B and Table S3, Supporting Information).^[^ ^24^ ^]^ Interestingly, we found that the ratio of exhausted T cells in the peripheral blood in this dataset, which was similar with our data, was higher than that in the bone marrow (Figure S3C, Supporting Information). Thus, T cells in the peripheral blood may provide a good window for studying T cell clusters and exhausted T cells’ signature in B‐ALL state.

### 2.2 Different T Cell Subtypes Prevalently Show More Activated Characteristics in B‐ALL

**Figure 2:**
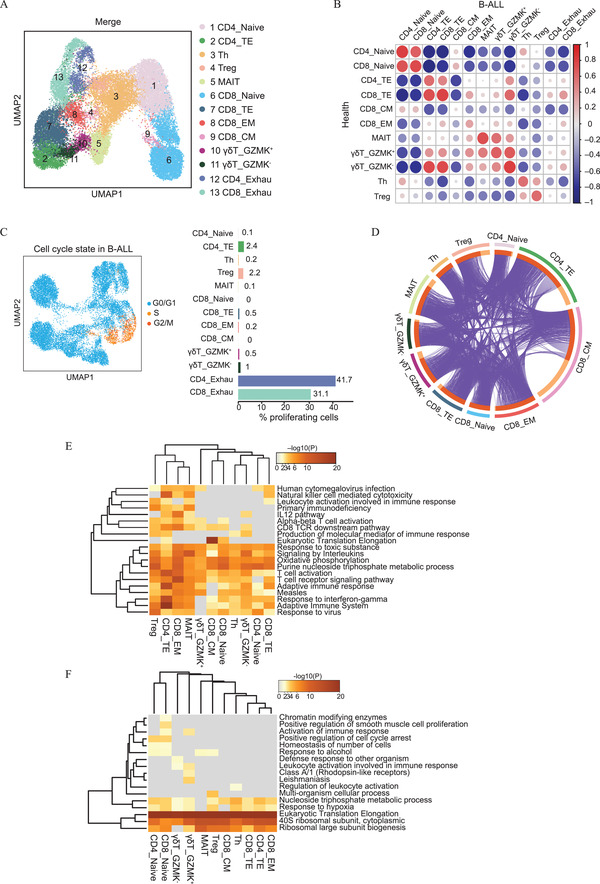
Integrated analysis of T cells in healthy individuals and B‐ALL patients. A) UMAP showing the merge of eleven and thirteen T cells from healthy individuals and B‐ALL patients. Each dot corresponds to one single cell colored according to cell cluster. B) Dot plot showing the similarity of the eleven and thirteen T cell clusters in healthy individuals and those in B‐ALL patients. The colors and sizes are scaled to the pairwise Pearson correlation coefficient. C) UMAP plots showing the three cell cycle phases of the thirteen T cell clusters in B‐ALL patients (left) with the percentage of proliferating cells in the different B‐ALL clusters (right). The percentages are shown to the right. D) Circos plot showing the relationships of overlapping DEGs among the indicated T cell clusters. DEGs were identified by comparing B‐ALL patients to healthy individuals for each cluster. The purple lines link genes shared by two clusters. Genes found on multiple lists are colored in dark orange, and genes unique to a list are shown in light orange. E,F) Heatmaps showing the top enriched terms by Metascape enrichment analysis for DEGs (upregulated pathways shown in (E), downregulated pathways shown in (F) identified by comparing matched clusters between healthy individuals and B‐ALL patients. The heatmap cells are colored by their *p* values, grey cells indicate the lack of enrichment for that term in the corresponding gene list. *P* value < 0.05 was considered statistically significant.

Next, we combined all T cells from healthy individuals and B‐ALL patients. As expected, 11 T cell clusters were identified in healthy individuals, and these clustered together with corresponding clusters in B‐ALL patients (**Figure** **2**A). Pairwise Pearson correlation analysis also revealed that paired T cell clusters under both conditions have similar expression patterns with the exception of distinct exhausted T cell clusters (Figure 2B). We further analyzed the cell cycle phase of the T cells in healthy individuals and B‐ALL patients (Figure S3D,E, Supporting Information). Cell cycle scores, which were previously shown to denote the G0/G1, S, and G2/M phases,^[^ ^22^ ^]^ were used to infer the cell cycle phase. In healthy individuals, the T cells largely remained in the G0/G1 state, while a significant fraction of T cells (≈20.4%) were observed to be cycling in the leukemia environment. Specifically, we found that exhausted clusters (CD4_Exhau and CD8_Exhau), which were enriched for exhaustion genes, were the highest proliferating populations (41.7% and 31.1%, respectively) (Figure 2C). Indeed, this result is consistent with recent research suggesting that exhausted T cells are the major proliferating immune cell compartment.^[^ ^23^ ^]^

However, the response of different T cell clusters to the occurrence of B‐ALL remains unclear. To explore transcriptome changes in the leukemia environment, we identified differentially expressed genes (DEGs) in each cluster pair by comparing the patients with leukemia to the healthy individuals (with the exception of exhausted T cells) and then performed gene functional enrichment analysis of the DEGs in the B‐ALL environment. We observed that the majority of upregulated genes were shared among T cell clusters, and similar biological processes were activated in B‐ALL patients (Figure 2D). The same result was found for downregulated genes (Figure S3F, Supporting Information). Upregulated gene classes included those involved in antigen presentation (*HLA‐DQA1* and *HLA‐DQB1*), T cell activation (*CD27* and *CTLA4*), cytotoxicity (*GNLY* and *GZMB*), oncogene activation (*RHOA* and *CBLB*), and the cytokine‐mediated signaling pathway (*IL2RG* and *IL7R*) (Figure 2E). The most significantly downregulated pathways involved ribosomal biogenesis (*RPLs* and *RPSs*) and the hypoxia response (*CYBA* and *ND4*) (Figure 2F).

Taken together, the T cells in B‐ALL displayed prevalent activation. This observation indicates that T cells in the leukemia state are remodeled to up‐regulate their antigen presentation and immunotoxicity with the aim of removing leukemia cells.

### 2.3 Transcriptomic Heterogeneity of Exhausted T Cells in B‐ALL Patients

As previously mentioned, the exhausted T cell clusters were patient specific and highly cycling. Because *PDCD1* and *LAG3* are also co‐inhibitory receptors and targets of immunotherapies, we focused further analyses on these cells.

**Figure 3:**
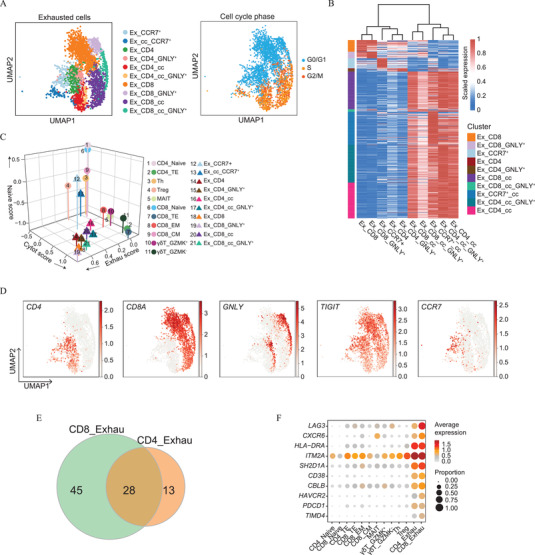
Dissection of exhausted T cell clusters in B‐ALL patients. A) UMAP showing 10 sub‐clusters of exhausted T cells (left). The plot to the right shows the cell cycle phases of the exhausted T cells. B) Heatmap showing expression of top 5 DEGs across clusters in the exhausted T cell clusters. C) 3D‐scatter plot illustrating three feature scores (naive, cytotoxicity, and exhaustion) for all T cell clusters. Each circle represents one T cell cluster, and each triangle represents an exhausted T cell cluster. D) UMAP plots showing the expression of five selected genes (*CD4*, *CD8A*, *GNLY*, *TIGIT*, and *CCR7*) in exhausted T cells. E) Venn diagram showing the overlap of presumed exhaustion marker genes identified by using CD8 exhausted cells (pale green) and CD4 exhausted cells (orange) compared to corresponding control cells. See Figure S3F, Supporting Information and Experimental Section for details. F) Dot plots show the average expression levels and cell expressing proportions of selected exhausted T cell signature genes in the indicated clusters. The colors represent the average expression levels, and dot sizes represent the percentage expression of selected genes in the indicated clusters.

We therefore examined the exhausted T cells in our single‐cell data. Unsupervised analysis further identified that exhausted CD4/CD8 T cells cluster into ten sub‐clusters (**Figure** **3**A,B). These sub‐clusters display exhaustion genes at different levels, and each appeared to be distinct when accounting for a combination of signatures (expression patterns) associated with the cell cycle status, naïve state, or *GNLY* expression (Figure 3A,C,D, Figure S4A, Supporting Information). As expected, all exhausted sub‐clusters exhibited significantly higher exhaustion scores compared with non‐exhausted clusters. Ex_CCR7^+^ and Ex_cc_CCR7^+^ exhibited higher naïve scores and relatively lower cytotoxic scores, while the latter was more cycling. Compared to the two canonical naïve clusters (CD4_Naive and CD8_Naive), Ex_CCR7^+^ was characterized by higher expression of genes associated with cytotoxicity, including *GZMK*, *GZMA*, and *NKG7* (Figure S4B, Supporting Information). Ex_CD4 and Ex_CD4_GNLY^+^, Ex_CD8 and Ex_CD8_GNLY^+^, Ex_CD4_cc and Ex_CD4_cc_GNLY^+^, and Ex_CD8_cc and Ex_CD8_cc_GNLY^+^ were distinguished from each other by the *GNLY* expression level (Figure 3C). The significance levels of the scores between two clusters are shown in Figure S4C, Supporting Information. Furthermore, gene set enrichment analysis (GSEA) was performed to compare the four exhausted GNLY^+^ clusters (Ex_CD4_GNLY^+^, Ex_CD8_GNLY^+^, Ex_CD4_cc_GNLY^+^, and Ex_CD8_cc_GNLY^+^). The results demonstrated that the clusters with *GNLY* expression were activated in the TCR signaling pathway, natural killer cell‐mediated cytotoxicity, and cytokine‐cytokine receptor interaction (Figure S4D, Supporting Information). Thus, we conclude that *GNLY* expression was positively correlated with a higher cytotoxic ability. Collectively, our data suggest that the exhausted T cell pool should be considered a heterogeneous cell compartment consisting of T cells in various states, possibly with different biological functions.

We then compared the exhausted CD8^+^ T cell cluster to the CD8_Naive, CD8_EM, CD8_CM, and CD8_TE clusters and the exhausted CD4^+^ T cell cluster to the CD4_Naive, CD4_Th, CD4_Treg, and CD4_TE clusters (Figure 3E/Figure S4E, Supporting Information). Finally, a list of 28 exhaustion‐specific genes was obtained. Known exhaustion markers, including *HAVCR2*, *PDCD1*, *LAG3*, and *HLA‐DRA*, were among the top‐ranked genes. The gene list also contained several less‐described genes including *CXCR6*, *ITM2A*, *SH2D1A*, *CD38*, *CBLB*, and *TIMD4* (Figure 3F). Based on The Cancer Genome Atlas acute myeloid leukemia (AML) data (https://www.cancer.gov), higher expression of *CXCR6*, *CD38*, and *CBLB* is associated with poor prognosis. Recent reports have shown that high expression of *ITM2A* is associated with poor prognosis in ovarian cancer,^[^ ^25^ ^]^ and *TIMD4* might provide a novel strategy for improving the clinical efficacy of cancer immunotherapy.^[^ ^26^ ^]^ Thus, our data not only confirm that previously identified genes are associated with exhausted T cells but also identify potential biomarkers for diagnosis and immunotherapy.

### 2.4 The TCR Repertoire Indicates Extensive T Cell Transition in Patients with B‐ALL

**Figure 4:**
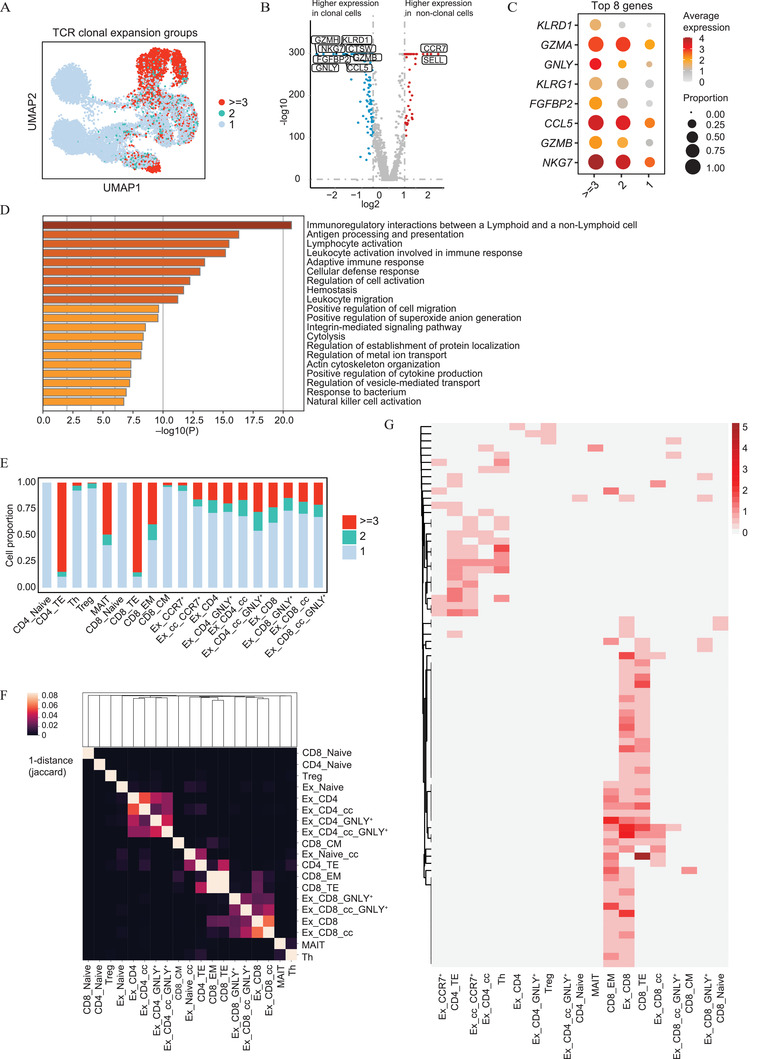
TCR repertoire analysis of T cells in B‐ALL patients. A) UMAP plot showing the concentration of clonal expansion in B‐ALL patients. The colors correspond to three TCR clonal expansion groups where “> = 3” indicates that there were at least three T cells that express identical TCR clonotype, “2” indicates that there were two T cells that express an identical TCR clonotype, and “1” indicates there was only one T cell that expresses a specific TCR clonotype. B) Volcano plot showing genes differentially expressed (see Table S3, Supporting Information) between highly expanded clones (“> = 3” group) and non‐clonal cells (“1” group). Highly expressed genes are shown in red, and genes with low expression are shown in blue. The top 10 most significant DEGs are also indicated. C) Dot plots show the average expression levels and cell expressing proportions of the top 8 highly expressed genes in the highly expanded group compared to the non‐clonal group. The colors represent the average expression levels, and dot sizes represent the number of expressed genes within a group. D) Top 20 most enriched terms for the up‐regulated DEGs in the highly expanded compared with non‐clonal group. The bars are colored by their p values, and *p* value < 0.05 was considered statistically significant. E) Bar charts showing the cellular proportion of three clonal expansion groups in each T cell cluster (including exhausted T cell sub‐clusters) in B‐ALL patients. F) Heatmap showing the Jaccard‐index between the respective pair of clusters in B‐ALL patients. For each tile, the brighter the color, the greater number of overlapping clonotypes between two corresponding clusters. G) Heatmap showing the distribution of shared clonotypes between exhausted and non‐exhausted T cell clusters in B‐ALL patients. Each row represents a shared clonotype and each column represents a T cell cluster. The colored squares are proportional to the number of cells with the same clonotype in the corresponding cluster.

Antigen recognition and clonal T cell expansion with clone‐specific TCRs are critical for adaptive immunity. Additionally, TCRs are often used as unique identifiers of T cell ancestries.^[^ ^27^ ^]^ To determine the extent to which clonal T cell expansion occurs in the B‐ALL state, we performed clonal analysis of T cells from B‐ALL patients (**Figure** **4**A/Table S4, Supporting Information). First, we profiled the V(D)J usage and length of the T‐cell receptor alpha gene complementarity determining region‐3 (TRA‐CDR3) and T‐cell receptor beta gene complementarity determining region‐3 (TRB‐CDR3) regions under the healthy and B‐ALL states (Figure S4A, Supporting Information). It is worth noting that there is discrepancy in the CDR3 length in the CD8_TE cluster between healthy individuals and patients with B‐ALL. The variable TCR clonotype composition of individual T cell clusters together with the differential expression of key genes suggests that phenotypic states are likely shaped by a combination of antigen TCR stimulation and environmental stimuli. As shown in Figure S5B/Table S4, Supporting Information, T cells in leukemia have more diverse clonotypes, and the population with highest clonality was mainly concentrated in the CD8_TE and CD4_TE populations, indicating abnormal activation of the immune system with leukemia antigen exposure.

Consistent with studies of other cancer types,^[^ ^28^, ^29^ ^]^ the majority of TCRs were unique. However, the TCR clonotype composition was highly variable among patients (Figure S5C, Supporting Information). We found that each patient was strongly dominated by a small number of T cell clones, indicating high clonal expansion of certain T cells. Furthermore, each cluster comprised different combinatorial subsets of clonotypes. Transcriptomic and TCR profiles of the same individual T cell provided us the opportunity to compare transcriptome differences between clonally expanded T cells and non‐clonal ones and dissect the clonotype distributions of the T cell clusters. Clonal expansion was observed with clonal sizes ranging from 2 to 273 (Table S4, Supporting Information). Clonally expanded cells were mostly in the G0/G1 phase and highly expressed the cytotoxic effector molecules granzymes B and H (*GZMB* and *GZMH*), *FGFBP2*, *GNLY*, *NKG7*, *KLRD1*, and chemokines (*CCL5*) (Figure 4B,C/Table S5, Supporting Information). Metascape enrichment analysis demonstrated that these cells are more engaged in antigen processing and presentation, lymphocyte activation, the immune response, and cellular defense pathways (Figure 4D). Generally, the naïve clusters CD4_Naive and CD8_Naive displayed very limited clonal expansion. CD8_CM, Th, and Treg clusters showed minimal clonal expansion. High clonal expansion was mainly focused in the CD4_TE, MAIT, CD8_TE, CD8_EM, and exhausted clusters (Figure 4E).

Pearson correlation analysis was performed to assess the relationship between the clonotype combinations in every cluster (Figure 4F). It was found that the four exhausted CD4^+^ clusters gathered together and the four exhausted CD8^+^ clusters also gathered together, indicating that they shared similar TCR signatures. As expected, a close relationship between CD8_EM and CD8_TE was found. Interestingly, these two clusters shared more clonotypes with exhausted CD8^+^ clusters than other T cell clusters. The number of clones shared between the exhausted clusters (observed as a whole population) and CD8_TE and CD8_EM was 30/2133 (1.41%) and 28/2133 (1.31%), respectively. Other T cell clusters also shared limited TCRs with the exhausted clusters (Figure 4G). Our data demonstrate that different clusters of T cells were by no means completely isolated, and they might undergo an extensive state transition. Furthermore, we fully speculated that exhausted CD8^+^ clusters, CD8_TE, and CD8_EM have an identical derivation, that is, the same as exhausted CD4^+^ clusters and CD4_TE.

## 3 Discussion

Among human immune cells, T cells recognize a broad range of antigens via a huge heterogeneous population of TCRs. TCRs vary from cell to cell and represent as “molecular tag” for T cells. Connecting TCR clonotype together with transcriptional landscape in individual T cells will be powerful for understanding the dynamics of clonal expansion within T cell populations.^[^ ^30^ ^]^ scRNA‐seq is a powerful technology for resolving heterogeneous cell types or states in human tissues and provides insights into cell functional heterogeneity. Nowadays, the application of this technique to immune profiling has begun to be realized.^[^ ^31^, ^32^ ^]^ Various scRNA‐seq methods were established. Among them, well‐based scRNA‐seq such as SMART‐seq2^[^ ^22^, ^33^ ^]^ and microfluidics‐enabled scRNA‐seq represented by Fluidigm C1^[^ ^34^, ^35^ ^]^ can achieve high sensitivity and get full‐length information including gene expression, splicing variants, and TCR repertoire diversity at the same time. However, these methods are expensive and time‐consuming for large‐scale experiments. Recently, 10X immune profiling provides a high‐throughput and commercial way; although it cannot get full‐length information and highly depend on cell quantity and activity, this strategy enables profiling thousands of genes at the 5′ end while simultaneously getting TCR repertoire from the same cells.^[^ ^12^ ^]^ Moreover, it has been well established for use in tumor‐related studies, such as lung tumor and breast tumor.^[^ ^12^, ^36^ ^]^

The tumor microenvironment resulting in cell immune suppression is a common characteristic of patients with tumors, particularly leukemia. Although clonally expanded T cells can also be identified in patients with leukemia, which is thought to be a specific response to leukemia‐associated antigens, such antigen‐specific T cells are incapable of eliminating leukemia cells due to their lower activity and higher expression of the exhausted phenotype.^[^ ^2^, ^37^ ^]^ Unlike the myeloid leukemia and other tumors which tumor associated antigens can induce either T cell or B cell immune response against tumor cells, patients in B‐ALL status are almost lacking B cell immune response including the B‐T cell interaction, due to the malignant clonal expanded B cells inhibit normal B cell proliferation. And many other immune cells secrete inhibitory cytokines or produce ROS.^[^ ^38^ ^]^ All these factors contribute to T cell hyper‐active and furtherly dysfunction. While T cell dysfunction affects the curative effects and prognosis of leukemia patients, increasing data have indicated that T cell immunotherapy such as CAR‐T cell therapy plays an important role in leukemia; however, the lower activation and terminal differentiation of T cells in patients limit its effects. CAR‐T cells prepared from dysfunctional T cells may have weakened function, barriers in cell proliferation, and persistence, leading to high recurrence rate after therapy.^[^ ^39^ ^]^ Thus, better understanding T cell clusters and their transcriptional profiles in leukemia patients may help to resolve this problem. Several recent studies have utilized scRNA‐seq to describe T cells in non‐small cell lung cancer (NSCLC), breast cancer, and colorectal cancer.^[^ ^12^, ^28^, ^36^, ^40^ ^]^ Intratumoral T cells greatly differ between tumors and are also highly heterogeneous within patients.^[^ ^30^ ^]^ In this study, we performed single‐cell profiling of T cells from peripheral blood samples of B‐ALL patients to characterize how the B‐ALL microenvironment affects T cells.

In previous single‐cell studies of B‐ALL and AML, T cell clusters did not appear to exhibit any consistent exhaustion transcriptional program in the bone marrow, which may attribute to insufficient clustering of T cells.^[^ ^24^, ^41^ ^]^ However, in our study, we observed and depicted the 11 classic T cell clusters in both healthy individuals and B‐ALL patients with two additional exhausted T cell clusters being patient specific. These clusters were comparable across samples. T cell exhaustion was previous characterized as a loss of proliferative capacity and high levels of PD‐1 and LAG3 expression in human tumors.^[^ ^28^, ^40^, ^42^ ^]^ Here, we showed that the exhausted T cell pool was not a discrete cell population but consisted of diverse states. Some of the exhausted T cells displayed naïve signatures with low cytotoxic scores, and other exhausted T cells demonstrated ongoing proliferation with relatively low exhaustion scores. It has been described that the naïve‐like exhausted T cells responsible for the proliferative burst after anti‐checkpoint therapy.^[^ ^43^ ^]^ We presumed that these cells may just begin to appear during the buildup of the exhausted program, whereas more advanced exhausted cells lost their proliferative signature as time went on. These results are in line with a previous observation in melanoma.^[^ ^11^ ^]^ In future work, tracking the dynamic shifts in these clusters throughout the course of B‐ALL progression would be imperative to establish rational immunotherapeutic interventions. Functional studies of the patient‐derived T cells on CAR‐T therapy are warranted to resolve their immunotherapy effect.

Our approach enabled the analysis of previously underappreciated subpopulations that were not detected by bulk RNA‐seq B‐ALL studies. We observed markedly high expression of genes related to cytotoxic activity (*NKG7*, *GNLY*, *GZMK*, and *GZMH*) in MAIT cells and *γδ* T cells. This finding is consistent with previous knowledge that MAIT and *γδ* T cells act as a first line of defense to kill leukemia cells.^[^ ^20^ ^]^ The roles of these genes in B‐ALL and immunotherapies need further investigation.

T cells display a broad continuum of active pathways in leukemia, which may be due to the stress response to leukemia antigens. T cells up‐regulate their antigen presentation and immunotoxicity to remove leukemia cells. TCR diversity may partially account for the T cell activation because the activated state was also found in polyclonal T cells.^[^ ^44^ ^]^ When analyzed in conjunction with TCR clonotypes, we could infer the characteristics of clonal expansion in each T cell cluster in the leukemia state. CD4_TE, MAIT, CD8_TE, CD8_EM, and exhausted clusters displayed a high level of clonal expansion. Importantly, shared TCR clones between the exhausted T cells and other T cells provided independent evidence for the existence of a differentiation trajectory. We speculate that exhausted CD8^+^ clusters may be derived from the CD8_TE and CD8_EM sub‐clusters, and exhausted CD4^+^ clusters may be derived from the CD4_TE sub‐cluster. In this way, TCR‐dependent trajectories for exhausted cells from TE cells suggest therapeutic strategies to lessen the conversion from TE cells to exhausted cells. Thus, our data support a model in which ongoing T cells activate toward exhaustion in the B‐ALL environment. However, the link between exhausted T cells and other T cells will have to be classified within a broader context of tumors.

Majority of clinical studies in leukemia focus on peripheral blood samples due to the easy accessibility. Furthermore, in clinical application, CAR‐T cell therapy usually adopts T cells from patients’ peripheral blood. Whereas, it has been known that mature T cells undergo extensive migration from the blood to the bone marrow and vice versa. Evidence shows memory T cells reside in the niche of bone marrow and persist over time in both healthy and leukemia station.^[^ ^45^, ^46^ ^]^ Being the important immunological environment, leukemia bone marrow is also thought to be the major influence site of T cell immunosuppression including increasing T cell exhaustion. Interestingly, we showed that the frequency of exhausted T cells in the peripheral blood was higher than that in the bone marrow in B‐ALL patients by computational re‐analyzing the scRNA‐seq datasets from a previous report.^[^ ^24^ ^]^ This finding highlights T cells in the peripheral blood as a good window for studying exhausted T cells’ signature. Moreover, the result also suggests the difference in immunological environment between bone marrow and peripheral blood in B‐ALL patients, although the underlying mechanisms remain elusive. Further investigation to compare the T cell profile including TCR repertoire between peripheral blood and bone marrow at the same time may well define this issue.

Collectively, this work revealed detailed characteristics of CD45^+^CD3^+^ T cells in B‐ALL in terms of their clustering, activity, and unique signatures. Exhausted T cells possess remarkable heterogeneity, and other T cell clusters activate and ultimately contribute to this compartment. Moreover, our characterization of T cells in the B‐ALL environment will facilitate a better understanding of the potential mechanisms underlying the effectiveness of immune‐based therapies targeting B‐ALL.

## 4 Experimental Section

### Experimental Samples

This project was approved by the human research ethics committee of Jinan university. All participating healthy individuals and patients provided written informed consent. The two healthy individuals had no tumors and other diseases on physical examination. The three patients were diagnosed with B‐ALL by three senior pathologists. Peripheral blood mononuclear cells (PBMCs) were obtained from patients prior to hormone treatment. The available clinical characteristics of the samples are summarized in Figure S1A, Supporting Information, with h1 and h2 as healthy individuals and p1, p2, and p3 as B‐ALL patients.

### Isolation of Lymphocyte Cells and Preparation of Single‐Cell Suspensions

Lymphocyte cells of PBMCs were isolated using Ficoll–Hypaque (Sigma Aldrich) solution according to the manufacturer's instructions. Briefly, 3 mL of fresh peripheral blood was collected in an EDTA anticoagulant tube and subsequently layered onto Ficoll. After centrifugation, lymphocyte cells remaining at the plasma‐Ficoll interface were carefully transferred to a new tube and wash twice with 1× PBS with 1% BSA. Lymphocyte cells were then re‐suspended with FACS sorting buffer (PBS supplemented with 1% BSA) for subsequent staining.

### Antibody Labeling and FACS

Cells were labeled with monoclonal antibodies for 30 min at 4 °C in FACS sorting buffer. The following antibodies were used: BV421‐conjugated anti‐human CD45 (BD Biosciences, 563879), FITC‐conjugated anti‐human CD3 (Biolegend, 300306), APC‐H7‐conjugated anti‐human CD4 (BD Biosciences, 560158), Percp‐cy5.5‐conjugated anti‐human CD8 (Biolegend, 344710). After staining, cells were washed once and resuspended in FACS sorting buffer. Cell sorting was performed using BD FACS Aria III. All samples were gated based on forward and side scatters, followed by exclusion of doublets. Expression levels of CD45, CD3, CD4, and CD8 were gated by their negative controls of unstained cells and positive controls of cells stained by each antibody. CD45^+^CD3^+^ T cells were sorted into 1.5 mL tubes containing 300 µL ice‐cold PBS with 1% BSA. The abundance of the relevant cell populations determined by testing the sorted cells again by FACS, reached more than 99%. Data analysis was performed using FlowJo V10 software (http://www.flowjo.com).

### scRNA‐seq and Data Processing

scRNA‐seq of gene expression and immune profiling was performed using the 10X Genomics Chromium Single Cell platform at 3“end or 5”end according to manufacturer's instructions. Raw reads were aligned to the hg19 human transcriptome (UCSC) and gene expression and TCR profiles were quantified using the “count” and “vdj” subcommands of CellRanger software package (version 3.0.1) with default parameters.

### Quality Control

A total of five 10×‐derived data (two healthy individuals and three B‐ALL patients) were obtained. First, quality controls were performed to filter low quality cells and low expression genes. DoubletDetetion^[^ ^47^ ^]^ (version 3.0) were employed to remove doublets. Only singlet cells with more than 800 genes and 2000 UMIs and less than 15% of reads mapped to mitochondrial genes were retained. Furthermore, the average expression value of *CD3D*, *CD3E*, *CD3G*, *CD247* genes was calculated as the average expression level of CD3, and then only cells with the average expression of CD3 greater than 0.5 were used for further analysis in this study. In addition, only genes expressed in more than 10 cells were kept. Finally, 12 699 cells in healthy individuals and 16 143 cells in B‐ALL patients were retained for downstream analyses.

### Analysis of Gene Expression Data

After quality control, UMI data was subjected to Scanpy^[^ ^48^ ^]^ (version 1.5.1) for analyses. Gene expression matrix was normalized using Scanpy's pp.normalize_total function (target sum = 1e^4^) and Scanpy's pp.log1p function. High Variable Genes (HVGs) were identified with parameters n_top_genes = 3000 using Scanpy's pp.highly_variable_genes function. Scanpy's pp.pca function was performed with parameters top 20 PCs. Then, Scanpy's pp.bbknn function was run to remove batch effects. Finally, to perform clustering analysis, Scanpy's tl.louvain function based on the louvain algorithm was used.

### Identification of DEGs

DEGs were identified using the Wilcox method implemented by “FindMarkers” or “FindAllMarkers” functions in Seurat (version 3.2.2).^[^ ^49^, ^50^ ^]^ Genes with fold change more than 1.5 and *adjusted p* value less than 0.05 were considered as DEGs. The DEGs were listed in the Supplementary Tables.

### Pearson Correlation Analysis

To reveal similarities between different T cell clusters within and between healthy dataset and diseased dataset, their Pearson correlation coefficients were calculated in the integrated PCA spaces. In detail, each cluster was represented as a centroid of single cells belonging to the cluster in the PCA space, by averaging vectors of those cells. Then Pearson correlation coefficients were calculated using these centroids, that were averaged vectors.

### Cell Cycle Analysis

A previously reported core gene set, consisting of 43 G1/S genes and 54 G2/M genes^[^ ^51^, ^52^ ^]^ was used to perform cell cycle analysis using Scanpy's tl.score_genes_cell_cycle function.

### Exhausted, Cytotoxic, Naive Feature Score

Well‐known T cell feature genes,^[^ ^12^, ^52^ ^]^ including 5 exhausted genes (*PDCD1*, *TIGIT*, *LAG3*, *HAVCR2*, *CTLA4*), 9 cytotoxic genes (*NKG7*, *CCL4*, *CST7*, *PRF1*, *GZMA*, *GZMB*, *IFNG*, *CCL3*, *FGFBP2*), and 4 naïve genes (*CCR7*, *TCF7*, *LEF1*, *SELL*) were used to calculate the feature scores using the Scanpy's tl.score_genes function. Significance level of the differences in the feature scores between different T cell clusters was evaluated using "Wilcox test" and false discovery rata (FDR) value less than 0.05 was considered to be statistically significant.

### Gene Functional Enrichment Analysis

Network enrichment analyses were performed using Metascape (http://metascape.org).^[^ ^53^ ^]^ To respectively compare the differential expression of Ex_CD4_GNLY^+^ versus Ex_CD4, Ex_CD4_cc_GNLY^+^ versus Ex_CD4_cc, Ex_CD8_GNLY^+^ versus Ex_CD8, Ex_CD8_cc_GNLY^+^ versus Ex_CD8_cc, GSEA^[^ ^54^ ^]^ analyses were performed using clusterProfiler^[^ ^55^ ^]^ R package (version 3.14.3) with default parameters.

### SingleR Analysis

SingleR^[^ ^16^ ^]^ analysis was performed to validate the clustering results. As reported, SingleR package used unbiased cell type recognition from scRNA‐seq data, by leveraging reference transcriptomic datasets of pure cell types to infer the cell of origin of each single cell independently. In this study, three reference datasets were used (HumanPrimaryCellAtlasData,^[^ ^56^ ^]^ BlueprintEncodeData,^[^ ^57^, ^58^ ^]^ NovershternHematopoieticData^[^ ^59^ ^]^) to predict T cell types of the identified clusters. The top 20 most similar cell types are displayed in the heatmaps.

### Integrated Analysis of Matthew's Datasets

In order to compare the proportion of exhausted T cells and memory T cells between peripheral blood and bone marrow, Matthew's datasets which were downloaded from GEO under GSE130116 (bone marrow dataset) and GSE134757 (peripheral blood dataset) were re‐analyzed. For the peripheral blood dataset, which utilized cell hashing method, samples were demultiplexed using the HTODemux function in Seurat,^[^ ^60^ ^]^ and only cells tagged as single were retained. Then, the bone marrow dataset consisting of seven B‐ALL patients at diagnosis and the peripheral blood dataset consisting of two B‐ALL patients at diagnosis were included for subsequent analyses. Quality control was performed to filter out low quality cells and low expression genes. Genes that were detected in fewer than three cells were filtered out. Only cells with more than 500 genes, 1500 UMIs, and less than 15% of reads mapped to mitochondrial genes were retained. Both datasets were separately processed using Seurat analysis procedure for dimension reduction and cell clustering, and then T cells, defined based on unsupervised clustering results and the expression level of *CD3* genes, were selected for the integrated analysis.

Seurat3 integration method^[^ ^50^ ^]^ based on identification of “anchors” between pairs of datasets was used to eliminate biological and technical batch effect of the samples and scRNA‐seq libraries. Then, the T cells extracted from Matthew's datasets, as described above, and the data were integrated using the Seurat3 integration method with top 2000 HVGs. The integration anchors were identified based on the first 20 dimensions. UMAP analysis and cluster detection were performed using the RunUMAP and FindClusters functions in Seurat. Finally, T cell subsets were determined based on clustering results and the expression of specific T cell markers.

### TCR Profiling Analysis

In this study, Scirpy^[^ ^61^ ^]^ package was used to analyze the TCR sequencing data from one healthy individual and three patients. In peripheral blood, the vast majority of T cells express TCRs composed of two chains, *α*, and *β*. Since the TCR on the surface of *γδ*T cells was a heterodimer composed of *γ* and *δ* chains (TCR*γδ*), those cells belonging to “*γδ* T” clusters were excluded. After categorized cells based on how many α and β chains they had using Scirpy’s tl.chain_pairing, the cells were classified as follows, “No TCR”, “Orphan beta”, “Orphan alpha”, “Single pair”, “Extra alpha” and “Extra beta”, “Two full chains” and “Multichain”. Only the cells with “Single pair,” “Extra alpha,” and “Extra beta” chains were included for the next analyses. Then clonotypes were defined using Scirpy's pp.tcr_neighbors and Scirpy's tl.define_clonotype_clusters functions based on amino‐acid sequence similarity. Clonotypes were grouped into three groups as “> = 3,” “2,” and “1,” based on how many T cells express identical TCR. In order to evaluate the similarity of TCR clonotypes between different clusters, a matrix featuring the abundance of clonotypes in each cluster was created by performing Scirpy's tl.repertoire_overlap function. Additionally, it also computes a (Jaccard) distance matrix of clusters as well as the linkage of hierarchical clustering. The distance matrix was shown as a heatmap, while clusters were reordered based on hierarchical clustering.

### Statistical Analysis

All statistical analyses were conducted in Python (version 3.6) and R (version 3.6.3). Heatmaps of pathway enrichment analysis and circos plots of overlapping DEGs were generated by using Metascape (http://metascape.org), and *p* value less than 0.05 was considered statistically significant.

## Conflict of Interest

The authors declare no conflict of interest.

## Author Contributions

X.W., Y.C., and Z.L. contributed equally to this work. X.W. performed the experiments, analyzed the data, and wrote the manuscript. Y.C. and Z.L. performed the bioinformatics analysis and assisted with the manuscript. B.H. helped with the experiments and flow cytometry. L.X. assisted with the data interpretation. J.L., Y.Lu, and X.Z. provided the samples. B.L., Y.Lan, and Y.Li conceived the study, designed the experiments, interpreted the results, wrote the paper, and oversaw the research project.

## Supporting information

Supporting Information

Supplemental Table 1

Supplemental Table 2

Supplemental Table 3

Supplemental Table 4

Supplemental Table 5

## Data Availability

The scRNA‐seq data generated in this study are deposited at the NCBI Gene Expression Omnibus (GEO) with accession codes GSE172158. All other included data in this study are available from the corresponding authors upon reasonable request. Code is available on reasonable requests.
