# Supplementary material for: Single‐Cell RNA‐Seq of T Cells in B‐ALL Patients Reveals an Exhausted Subset with Remarkable Heterogeneity
Source: Adv Sci (Weinh). 2021 Aug 8;8(19):2101447. doi: 10.1002/advs.202101447 (PMC8498858; doi:10.1002/advs.202101447)
Supplement: Supplementary file 1 — Supporting Information [file ADVS-8-2101447-s002.pdf]

© 2021 Wiley-VCH GmbH

ADVANCED SCIENCE
Open Access

Supporting Information

for *Adv. Sci.*, DOI: 10.1002/advs.202101447

Single-cell RNA-seq of T cells in B-ALL patients reveals an exhausted subset with remarkably heterogeneity

Xiaofang Wang[1, 2#], Yanjuan Chen[2#], Zongcheng Li[3#], Bingyan Huang[2], Ling Xu[1, 2], Jing Lai[1], Yuhong Lu[1], Xianfeng Zha[4], Bing Liu[3], Yu Lan[2], Yangqiu Li[1, 2*]*

Supporting Information

Single-cell RNA-seq of T cells in B-ALL patients reveals an exhausted subset with remarkably heterogeneity

Xiaofang Wang[1, 2#], *Yanjuan Chen*[2#], *Zongcheng Li*[3#], *Bingyan Huang*[2], *Ling Xu*[1, 2], *Jing Lai*[1], *Yuhong Lu*[1], *Xianfeng Zha*[4], *Bing Liu*[3], *Yu Lan*[2*], *Yangqiu Li*[1, 2*]

[1]Department of Hematology, First Affiliated Hospital, Jinan University, No.601 West of Huangpu Avenue, Guangzhou 510632, China

[2]Key Laboratory for Regenerative Medicine of Ministry of Education, Institute of Hematology, School of Medicine, Jinan University, Guangzhou 510632, China

[3]State Key Laboratory of Experimental Hematology, Institute of Hematology, Fifth Medical Center of Chinese PLA General Hospital, Beijing 100071, China.

[4]Department of Clinical Laboratory, First Affiliated Hospital, School of Medicine, Jinan University, No.601 West of Huangpu Avenue, Guangzhou 510632, China

[#]These authors contributed equally to this work.

[*]Correspondence:

Yu Lan, rainyblue_1999@126.com

Yangqiu Li, yangqiuli@hotmail.com

Supporting information includes five supplemental figures, supplemental figure legends.

Figure S1

A

Sample ID	Age	Gender	WBC at diagnose	Immune type	Genetic information	Chemotherapy reaction	Time to get CR	Prognosis stratification	Cell number after quality control (with clonotype)
h1	29	Female	-	-	-	-	-	-	8562 (NA)
h2	35	Male	-	-	-	-	-	-	4107 (2977)
p1	55	Female	11.2*10^9	B	ABL1 copy number increase; TCF3/PBX1(+)	good	45 days	high	5060 (4419)
p2	23	Male	>50*10^9	B	CRLF2 copy number increase	good	30 days	high	6242 (3624)
p3	26	Male	1.56*10^9	B	-	good	45 days	intermediate	4841 (3675)

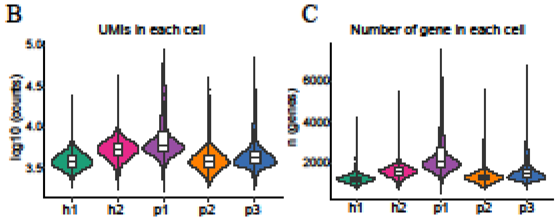

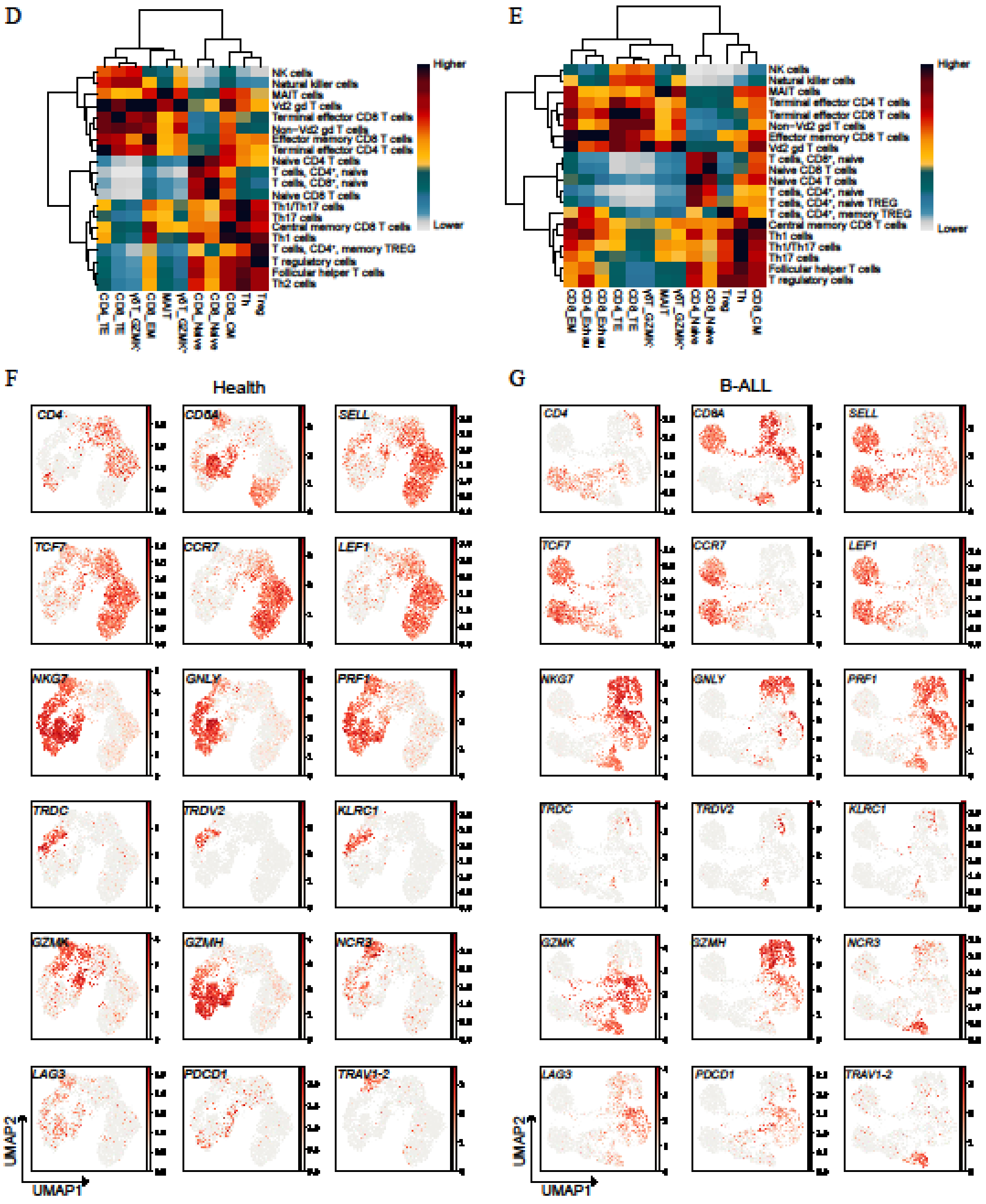

Figure S1. Sample information and transcriptomic features of the T cell clusters identified in healthy individuals and B-ALL patients.

A. Demographic information of the five samples used for scRNA-seq in this study.

B. Violin plots with boxplot insets showing the number of UMIs in each cell for each sample. The box plot in the violin shows the 25%, 50%, and 75% percentiles respectively.

C. Violin plots with boxplot insets showing the number of genes in each cell for each sample. The box plot in the violin shows the 25%, 50%, and 75% percentiles respectively.

D-E. Heatmap of the annotation scores of the T cell clusters in the healthy individuals (**D**) and B-ALL patients (**E**) derived with reference to the HumanPrimaryCellAtlasData, BlueprintEncodeData and NovershternHematopoieticData databases. Only labels (rows) with the top 20 highest scores in the results are shown in the plot. The darker the color, the more similar the two cell subtypes.

F-G. UMAP plots showing the expression of selected T cell marker genes in healthy individuals (**F**) and B-ALL patients (**G**).

Figure S2

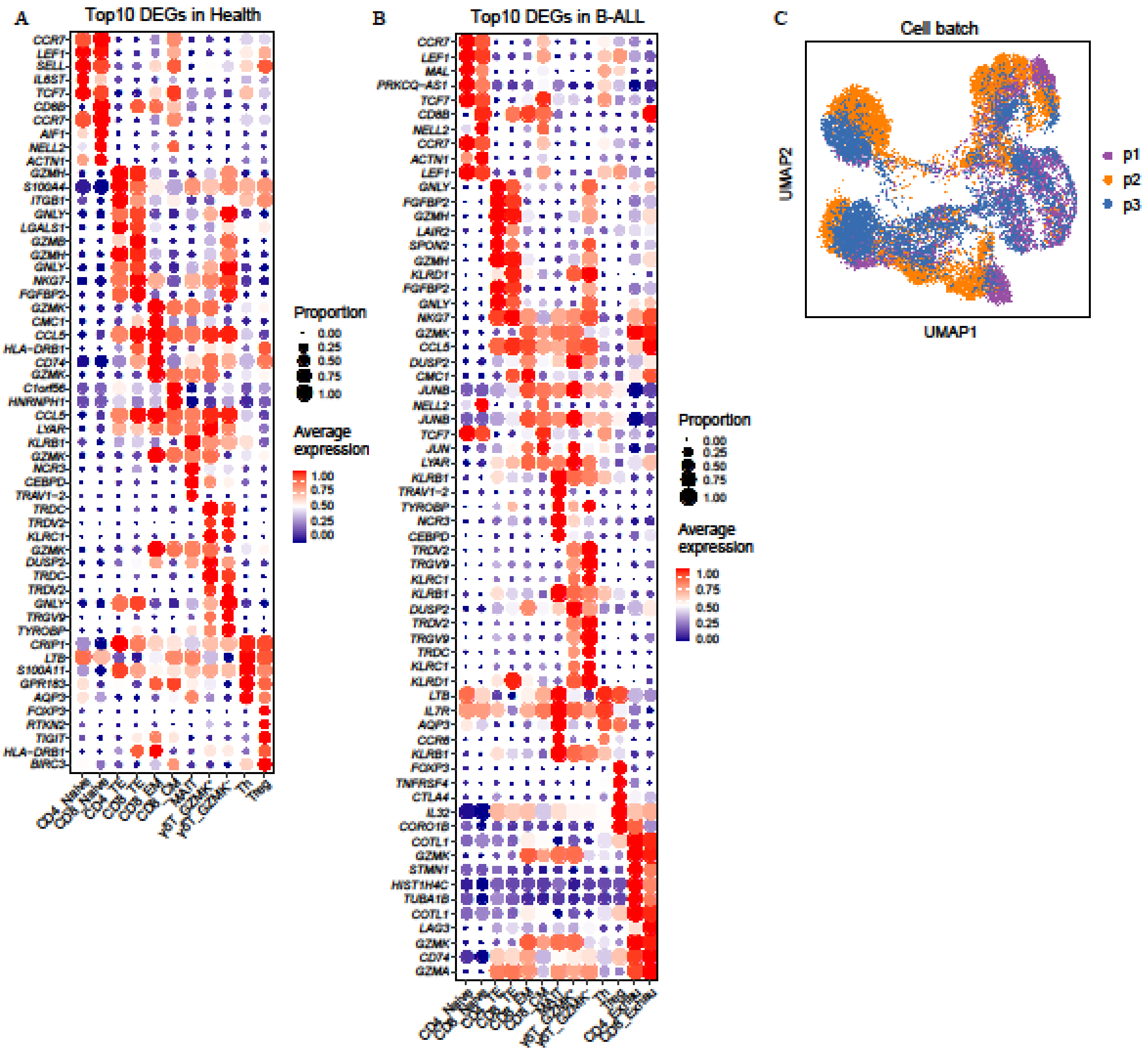

Figure S2. Comparison of DEGs in healthy individuals and B-ALL patients.

A-B. Dot plots show the average expression levels and cell expressing proportions of the top 5 differentially expressed genes (DEGs) across clusters in healthy individuals

(A) and B-ALL patients (**B**). The colors represent the average expression levels, and dot sizes represent the number of expressed genes within a cluster.

C. UMAP plot showing the cell batch information of three B-ALL patients. The colors represent each sample.

Figure S3

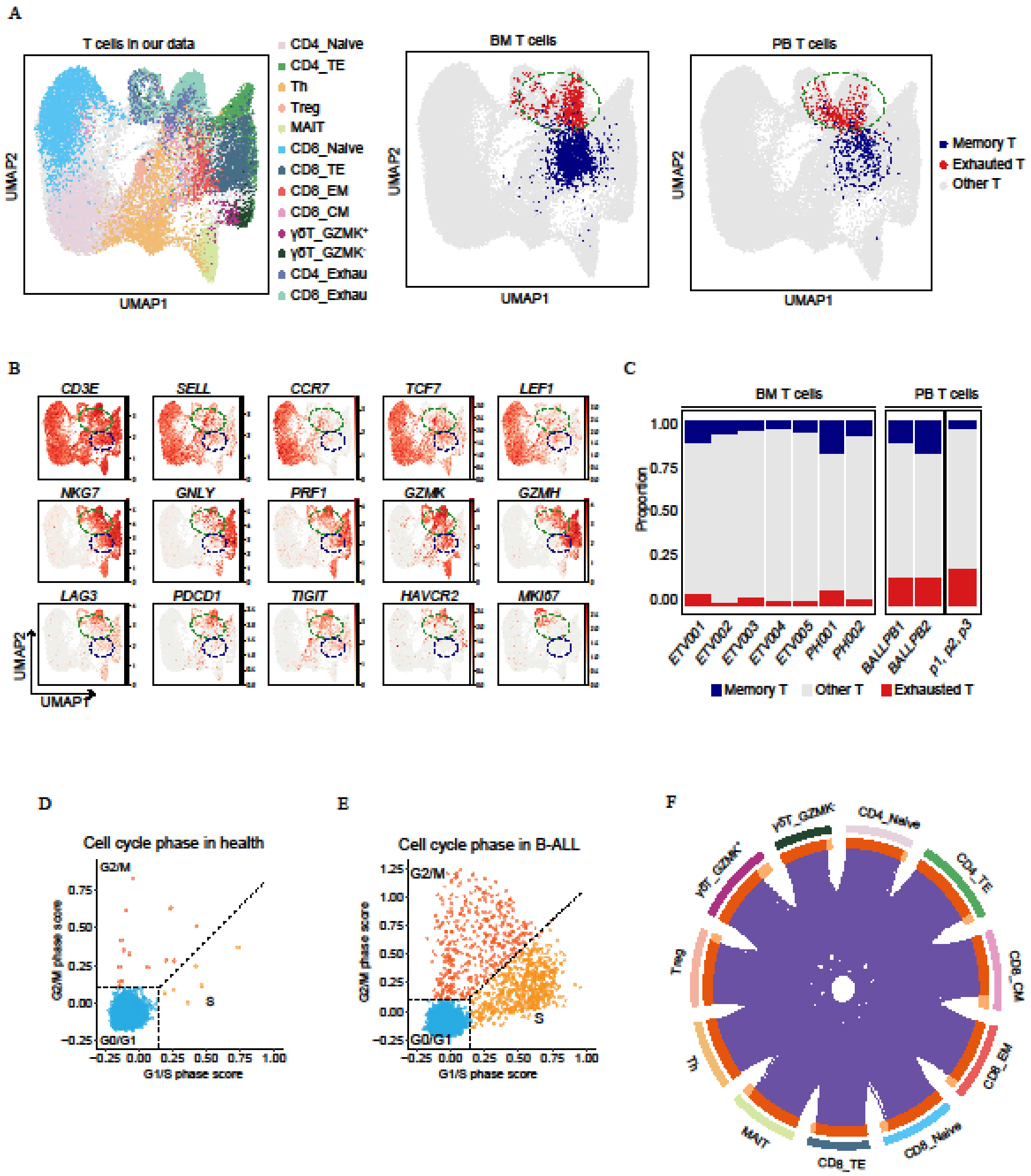

Figure S3. Integrated analysis of T cells in the Matthew's data and our study and comparison of cell cycle in healthy individuals and B-ALL patients

A. UMAP plot showing integrated T cell clusters of Matthew's data and our data (40,282 cells in total). The left UAMP plot shows the T cell clusters (including thirteen clusters as Figure 1B right plot) of B-ALL patients in our study, other T cells from Matthew's data are in grey. The middle UAMP plot shows the T cell clusters ("Memory T", "Exhausted T" and "Other T") of peripheral blood (2,737 cells in total) from Matthew's data. The right UAMP plot shows the T cell clusters ("Memory T", "Exhausted T" and "Other T") of bone marrow (21,402 cells in total) from Matthew's data. Each dot corresponds to one single cell, colored according to cell clusters.

B. UMAP plots showing the expression of selected T cell marker genes in integrated T cell clusters.

C. Stacked bar chart showing the constitution of T cell clusters ("Memory T", "Exhausted T" and "Other T") in each bone marrow sample from Matthew's data (abbreviated as "ETV001", "ETV002", "ETV003", "ETV004", "ETV005" and "PH001", "PH002"), each peripheral blood sample from Matthew's data (abbreviated as "BALLPB1" and "BALLPB2") and peripheral blood samples from our data (p1, p2, and p3).

D-E. Scatterplot showing the cell cycle scores of cells from the healthy individuals (**D**) and B-ALL patients (**E**). Each dot corresponds to one single cell colored according to cell cycle phases.

F. Circos plot showing the overlapping relationships of DEGs among the indicated T cell clusters. DEGs were identified by comparing patients to healthy individuals for each cluster. The purple lines link the same gene, which is shared by two clusters. Genes found on multiple lists are colored in dark orange, and genes unique to a list are shown in light orange.

Figure S4

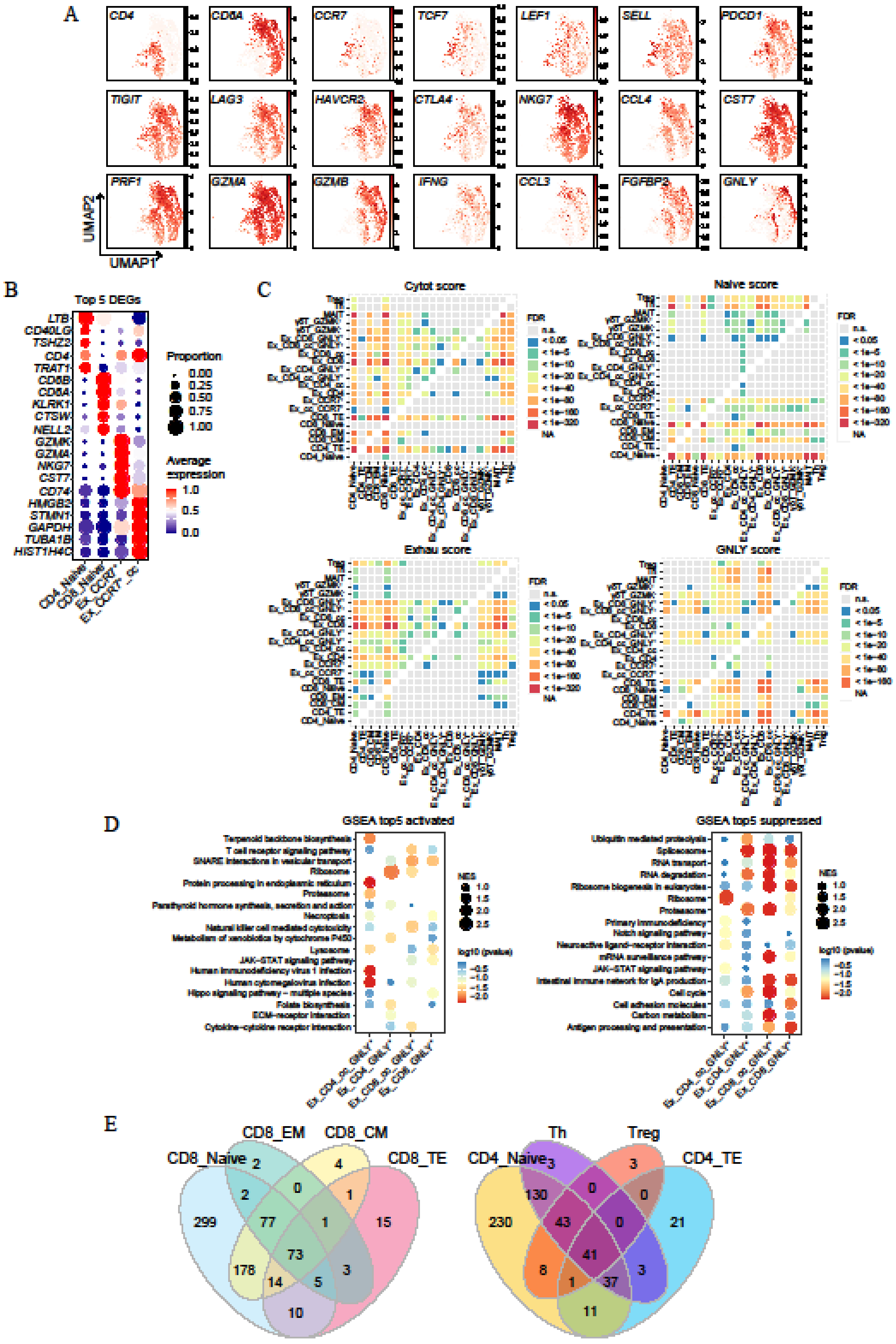

Figure S4. Transcriptomic characteristics of exhausted T cell sub-clusters in the B-ALL patients.

A. UMAP plots showing the expression of selected T cell marker genes in exhausted T cells.

B. Dot plots show the average expression levels and cell expressing proportions of the top 5 DEGs in the CD4_Naive, CD8_Naive, and Ex_CCR7$^+$ clusters. The colors represent the average expression levels, and dot sizes represent the number of expressed genes within a cluster.

C. Heatmaps showing the pairwise significance level for cytotoxic, naïve, exhausted, and GNLY-anchored scores between two clusters. Significance level was evaluated using Wilcox test. FDR<0.05 was considered to be statistically significant and FDR value> 0.05 was considered to be n.s. (no significance).

D. Dot plots showing the top 5 activated (left) and suppressed (right) signaling pathways in the four exhausted T cell clusters. GSEA analyses were performed by comparisons including Ex_CD4_GNLY$^+$ versus Ex_CD4, Ex_CD4_cc_GNLY$^+$ versus Ex_CD4_cc, Ex_CD8_GNLY$^+$ versus Ex_CD8, and Ex_CD8_cc_GNLY$^+$ versus Ex_CD8_cc.

E. Venn diagram showing the overlap of DEGs between exhausted T cells and each of the other non-exhausted clusters as indicated in the B-ALL patients. The left shows overlapping DEGs generated by comparing exhausted CD8$^+$ T cell clusters (considering the impact of the cell cycle, Ex_CD8_cc was not included) to CD8_Naive, CD8_EM, CD8_CM, and CD8_TE. The right shows overlapping DEGs generated by comparing exhausted CD4$^+$ T cell clusters (considering the impact of the cycle, the Ex_CD4_cc and Ex_CD4_cc_GNLY$^+$ clusters were not included) to CD4_Naive, CD4_Th, CD4_Treg, and CD4_TE.

Figure S5

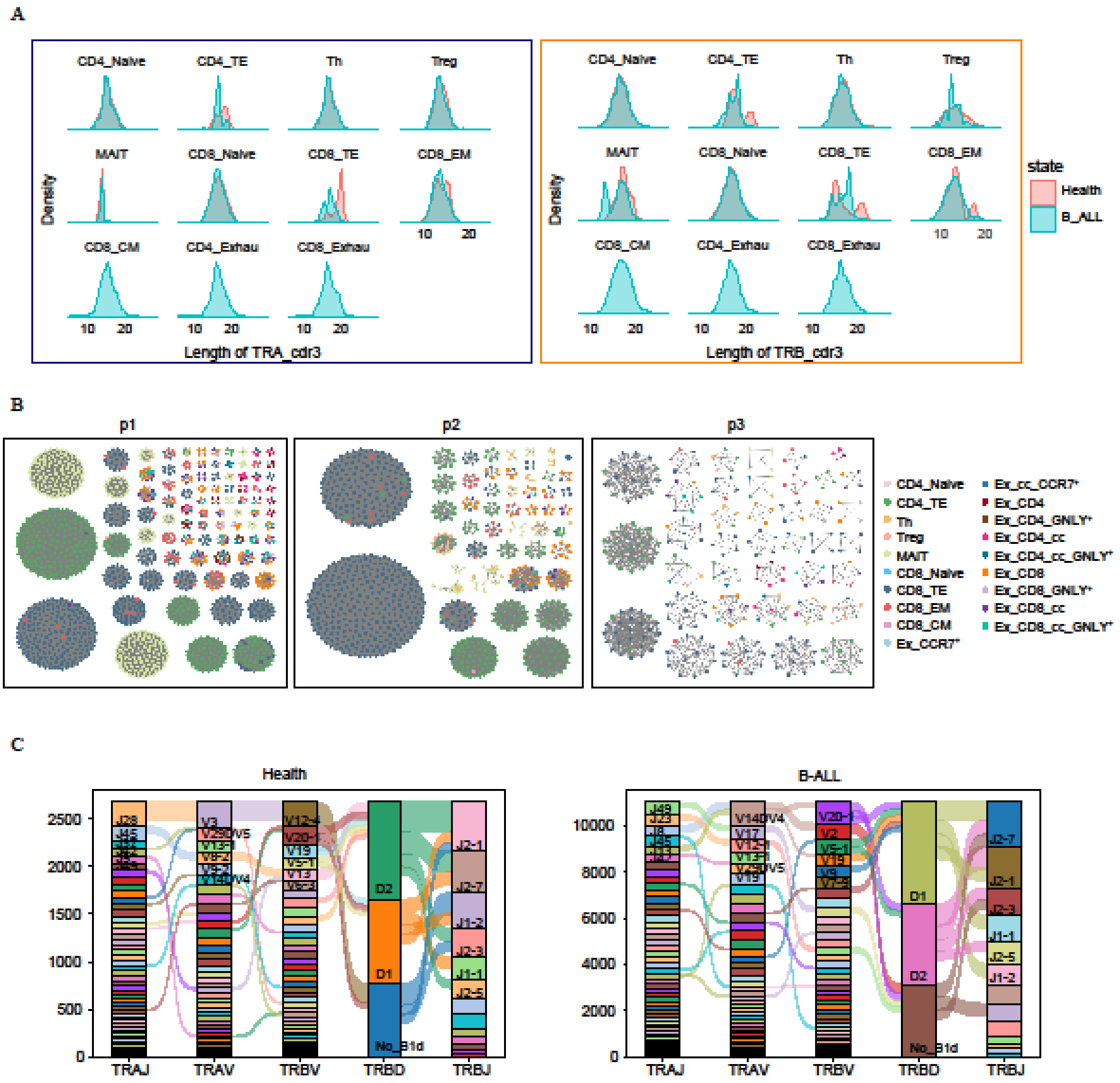

Figure S5. Characteristics of the TCR clonotypes in the healthy individuals and B-ALL patients.

A. Length of the TRA-CDR3 region amino acid chains (top) in the HIs and TRB-CDR3 region chains (bottom) in the B-ALL patients.

B. The networks of the TCR clonotypes in three B-ALL patients. Clonotype clusters with greater than 4 cells were visualized in a network plot. Each dot corresponds to a single cell and is colored according to cell cluster. Each convergent clonotype cluster represents a sub-network corresponding to a clonotype cluster defined based on amino acid sequence similarity. In each sub-network, the line between each dot is calculated based on the nucleotide sequence. Colors represent different cell sub-clusters.

C. Top 10 V(D)J gene usage of primary TCR α and β chains in the healthy individuals (top) and B-ALL patients (bottom) as visualized in a Sankey plot. Each bar refers to a specific V(D)J segment, and each bar section to a specific V, D, or J gene. Colors represent different V, D, or J gene.
